# Safety Endpoints With Vadadustat Versus Darbepoetin Alfa in Patients With Non**–**Dialysis-Dependent CKD: A Post Hoc Regional Analysis of the PRO_2_TECT Randomized Clinical Trial of ESA-Treated Patients

**DOI:** 10.1016/j.xkme.2023.100667

**Published:** 2023-05-12

**Authors:** Patrick S. Parfrey, Steven K. Burke, Glenn M. Chertow, Kai-Uwe Eckardt, Alan G. Jardine, Eldrin F. Lewis, Wenli Luo, Kunihiro Matsushita, Peter A. McCullough, Todd Minga, Wolfgang C. Winkelmayer

**Affiliations:** 1 Department of Medicine, Memorial University, St John's, Newfoundland, Canada; 2 Akebia Therapeutics, Inc., Cambridge, MA; 3 Stanford University School of Medicine, Palo Alto, CA; 4 Department of Nephrology and Medical Intensive Care, Charité–Universitätsmedizin Berlin, Berlin, Germany; 5 Institute of Cardiovascular and Medical Sciences, University of Glasgow, Glasgow, UK; 6 Department of Epidemiology, Johns Hopkins Bloomberg School of Public Health, Baltimore, MD; 7 Cardiorenal Society of America, Phoenix, AZ; 8 Section of Nephrology, Baylor College of Medicine, Houston, TX

**Keywords:** Anemia, chronic kidney disease, hypoxia-inducible factor, vadadustat, darbepoetin alfa

## Abstract

**Rationale & Objective:**

In the PRO_2_TECT trials, vadadustat was found to be noninferior to darbepoetin alfa in hematologic efficacy but not for major adverse cardiovascular events (MACE; all-cause death or nonfatal myocardial infarction or stroke) in patients with non–dialysis-dependent chronic kidney disease (NDD-CKD). We investigated the regional differences in MACE in the PRO_2_TECT trials.

**Study Design:**

Phase 3, global, open-label, randomized, active-controlled clinical trial.

**Setting & Participants:**

A total of 1,725 erythropoiesis-stimulating agent (ESA)-treated patients with anemia and NDD-CKD.

**Intervention:**

1:1 randomization to receive vadadustat or darbepoetin alfa.

**Outcomes:**

The primary safety end point was the time to first MACE.

**Results:**

At baseline, patients in Europe (n=444) were primarily treated with darbepoetin alfa, showed higher proportions on low ESA doses (<90 U/kg/wk epoetin alfa equivalents) with a hemoglobin concentration of ≥10 g/dL compared with patients in the US (n=665) and non-US/non-Europe (n=614) regions. The MACE rates per 100 person-years in the 3 vadadustat groups across regions were 14.5 in the US, 11.6 in Europe, and 10.0 in the non-US/non-Europe groups, whereas event rates in the darbepoetin alfa group were considerably lower in Europe than in the US and non-US/non-Europe groups (6.7 vs 13.3 and 10.5, respectively). The overall hazard ratio for MACE for vadadustat vs darbepoetin alpha was 1.16; 95% CI, 0.93-1.45, but varied by geographical region, with a greater hazard ratio seen in Europe (US, 1.07; 95% CI, 0.78-1.46; Europe, 2.05; 95% CI, 1.24-3.39; non-US/non-Europe, 0.91; 95% CI, 0.60-1.37); interaction between study treatment and geographical region, *P* = 0.07). In Europe, ESA rescue was associated with a higher risk of MACE in both groups.

**Limitations:**

Several analyses are exploratory.

**Conclusions:**

In this trial, there was a low risk of MACE in the darbepoetin alfa group in Europe. Patients in Europe were generally on low doses of ESA, with hemoglobin already within target range. The low risk of MACE may have been related to a limited need to switch and titrate darbepoetin alfa compared with the non-US/non-Europe group.

**Funding:**

Akebia Therapeutics, Inc.

**Trial Registration:**

ClinicalTrials.gov identifier: NCT02680574

## Graphical abstract

**Figure:**
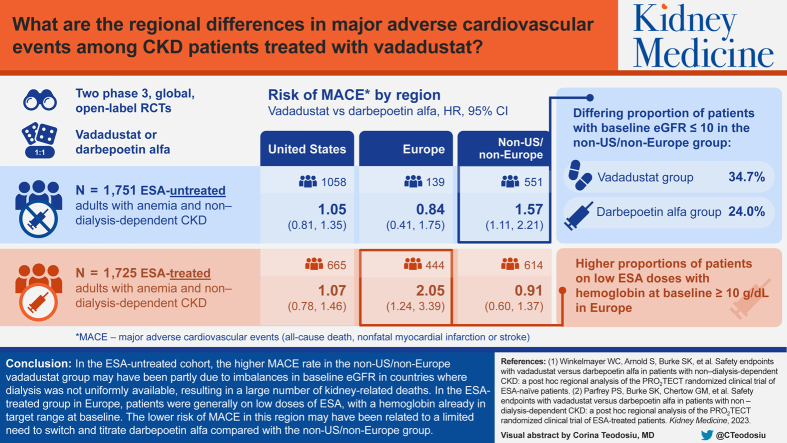

**Plain Language Summary**

Anemia is a common complication of chronic kidney disease (CKD), associated with reduced quality of life and heightened risk of cardiovascular events. Vadadustat is an investigational oral therapy for treatment of anemia due to CKD. In global phase 3 trials (PRO_2_TECT), vadadustat showed noninferiority to the injectable erythropoiesis-stimulating agent (ESA) darbepoetin alfa for time to first major adverse cardiovascular events (MACE) for patients with non–dialysis-dependent CKD. When analyzed by geographic regions, a notably low risk of MACE was seen among patients receiving darbepoetin alfa in Europe. These patients were on low doses of ESAs with close-to-target hemoglobin concentration levels before starting the trial, which could account for their lowered risk of MACE compared with other populations.

More than half of patients with advanced chronic kidney disease (CKD; stages 4 and 5) have anemia^1^^,^^2^ that is generally managed using oral and intravenous (IV) iron, erythropoiesis-stimulating agents (ESAs), and blood transfusions.^3^^,^^4^ Although these treatments are well accepted, treatment of anemia associated with non–dialysis dependent CKD (NDD-CKD) is variable among and within geographic regions and individual countries.^2^, ^3^, ^4^ These differences stem at least in part from the ambiguity of clinical practice guidelines and differences in reimbursement models.^5^ Regional differences in treatment behaviors and algorithms have influenced cardiovascular outcomes in clinical trials.^6^, ^7^, ^8^, ^9^

A new class of compounds for the treatment of anemia in CKD is the hypoxia-inducible factor prolyl hydroxylase inhibitors (HIF-PHIs), orally administered agents that work by stabilizing HIF, which in turn stimulates endogenous erythropoietin production and iron mobilization. The hematological efficacy and safety of the HIF-PHI vadadustat was recently compared with the ESA darbepoetin alfa in 2 global phase 3 trials in ESA-treated and ESA-untreated patients with NDD-CKD and anemia (PRO_2_TECT trials).^10^ Vadadustat was found to be noninferior to darbepoetin alfa with respect to hematologic efficacy but did not meet the noninferiority threshold for major adverse cardiovascular events {MACE; prespecified noninferiority margin for MACE of 1.25 (US Food and Drug Administration; [FDA]) and 1.3 [European Medicines Agency]}.^10^^,^^11^ However, in prespecified analyses by stratified geographic regions (US vs non-US groups), we observed a striking difference in MACE among patients randomized to vadadustat or darbepoetin alfa.^10^ In these analyses, we aim to further investigate the potential reasons for these regional differences in the trial of patients with NDD-CKD and anemia previously treated with ESA.

## Methods

We conducted 2 phase 3, global, open-label (sponsor-blinded), randomized, active-controlled clinical trials, collectively known as the PRO_2_TECT trials, comparing the administration of vadadustat with darbepoetin alfa in patients with NDD-CKD who were untreated (ClinicalTrials.gov identifier: NCT02648347) or treated (ClinicalTrials.gov identifier: NCT02680574) with ESAs. Trial sites were in North America, Latin America, Europe, Africa, and the Asia-Pacific region. Both trials were conducted in compliance with the International Conference on Harmonisation, in accordance with Good Clinical Practice guidelines and FDA regulations, and in line with the principles of the Declaration of Helsinki. We obtained institutional review board approval at each participating center, and all patients provided written informed consent before enrollment. The protocol and primary (pooled) results for these trials have been reported elsewhere.^10^^,^^11^

This analysis is limited to patients in the PRO_2_TECT trials who were actively maintained on ESA treatment at study entry (ESA treated), with at least one ESA dose received within 6 weeks before or during screening (Conversion trial; ClinicalTrials.gov identifier: NCT02680574). A second concurrent article reports results from the trial of patients who were ESA untreated at study entry.^12^ Eligible patients in this trial were adults with an estimated glomerular filtration rate (eGFR) of ≤60 mL/min/1.73 m^2^, hemoglobin concentration of 8-11 g/dL in the US sites or 9-12 g/dL at non-US sites, a serum ferritin concentration of ≥100 ng/mL, and transferrin saturation of ≥20%. We excluded patients who presented with anemia due to any known cause other than CKD; a red blood cell transfusion within 8 weeks before randomization; active malignancy or a history of active malignancy within 2 years; a recent cardiovascular or thromboembolic event; serum concentrations of alanine aminotransferase, aspartate transaminase, or total bilirubin >2 × upper limit of normal; or uncontrolled hypertension. The eligibility criteria did not include a lower limit for eGFR; however, patients were excluded if they were expected to start dialysis within 6 months from screening.

We randomized eligible patients 1:1 to receive vadadustat or darbepoetin alfa, stratified by region (US vs Europe vs non-US/non-Europe groups; see Table S1 for a list of countries that were finalized at the time of trial inception), New York Heart Association (NYHA) heart failure class (0/I vs II/III), and hemoglobin concentration at entry (<10.0 vs ≥10.0 g/dL). Oral vadadustat was administered at a starting dose of 300 mg once daily, with doses of 150 mg, 450 mg, and 600 mg available for adjustment to a maximum dose of 600 mg daily to meet the target hemoglobin concentrations. Darbepoetin alfa was dosed subcutaneously or intravenously based on the locally approved product label for patients with NDD-CKD. Patients receiving darbepoetin alfa at baseline were to continue darbepoetin alfa at the same dose, and those receiving epoetin alfa, epoetin beta, or methoxy polyethylene glycol-epoetin beta were converted to an equivalent darbepoetin alfa dose using standard conversion ratios.

The primary safety end point in the prespecified pooled analysis of the 2 PRO_2_TECT trials was time to first adjudicated MACE, defined as a composite of death from any cause, nonfatal myocardial infarction (MI), or nonfatal stroke. Secondary safety end points included time to first expanded MACE (defined as MACE plus hospitalization for heart failure or thromboembolic event, excluding vascular access thrombosis), cardiovascular (traditional) MACE, cardiovascular mortality, and all-cause mortality.

We conducted analyses on the population of patients who received ≥1 dose of trial drug (the safety population) and were stratified by prespecified regions (US vs Europe vs non-US/non-Europe groups).^10^

We assessed treatment-emergent adverse events (TEAEs) according to the Medical Dictionary for Regulatory Activities (MedDRA) version 23.0. Certain predefined TEAEs were sent to the MACE adjudication committee. Only the positively adjudicated MACEs were analyzed. All analyses were conducted using the SAS version 9 (SAS Institute Inc). The primary safety analysis was based on all first events that accrued across the 2 NDD-CKD trials (in ESA-untreated and ESA-treated patients). The sample size regarding the MACE end point was determined based on the number of events needed to show noninferiority based on the 2-sided 95% CI for the hazard ratio (HR) (vadadustat/darbepoetin alfa). We calculated that 631 events would be required overall (in both trials combined) to have 80% power to establish noninferiority with a margin of 1.25, and >90% power to establish noninferiority with a margin of 1.3, assuming no differences between the treatment groups. Since this trial and analyses reported only data from the PRO_2_TECT ESA-treated NDD-CKD trial, a smaller number of events and wider CIs for HRs were expected. A MACE rate of 10% annually was anticipated in both treatment groups based on a comprehensive review of available epidemiology and prospective clinical studies in the field.

An analysis of time to first MACE and expanded MACE were performed using a multivariable Cox regression model, including addition of the covariates of baseline hemoglobin, randomization strata of regions (US, Europe, and non-US/non-Europe groups) and NYHA class (0 or I, II, or III), sex (male or female), age (>65 or ≤65 years), race (White or non-White), and preexisting cardiovascular disease (yes/no) and diabetes mellitus (yes/no). Post hoc subgroup analyses reported here explored (1) MACE in patients receiving darbepoetin alfa at baseline compared with those receiving another ESA at baseline (ie, maintaining darbepoetin alfa vs switching from a different ESA), (2) MACE when adjusting for several baseline characteristics not included in the prespecified primary analysis (eg, baseline ESA dose [≤90 U/kg/wk, >90 and <300 U/kg/wk, and ≥300 U/kg/wk], baseline ESA type, urine albumin-to-creatinine ratio [uACR]), (3) differences in time to MACE as a function of the interaction between region and treatment, and (4) rescue defined as any exogenous ESA in the vadadustat group or another ESA in the darbepoetin alfa group or any dose increase of darbepoetin alfa ≥100% from the previous dose (≥4-fold the recommended 25% maximum dose increase).

Results for average weekly dose of the study treatment by region are reported as mean (SD), median, and lower and upper quartiles.

## Results

In total, 1,725 patients were randomized in the PRO_2_TECT trial that enrolled ESA-treated patients, and 1,723 patients received at least one dose of the study drug (safety population), which comprised 665 patients in the United States, 444 in Europe, and 614 outside the United States and Europe. The baseline demographic, clinical, and laboratory characteristics stratified by region are shown in Table 1. The European population was composed almost entirely of White patients, whereas the US population had approximately 29% Black patients and the non-US/non-Europe group included 16% Asian patients. Europe had a lower proportion of patients with diabetes, and more patients received IV iron compared to the US and non-US/non-Europe groups. Non-US/non-European patients were younger and had a lower prevalence of cardiovascular disease than patients in the United States or Europe. The mean baseline hemoglobin concentrations were lower in US patients (9.8-9.9 g/dL) compared with those in the Europe and non-US/non-Europe groups (10.7-10.9 g/dL).

**Table 1:** Selected Baseline Demographics, Clinical, and Laboratory Characteristics in ESA-Treated Patients (Safety Population)

Characteristic	US		Europe		Non-US/non-Europe	
	Vadadustat	Darbepoetin alfa	Vadadustat	Darbepoetin alfa	Vadadustat	Darbepoetin alfa
	(n=330)	(n=335)	(n=224)	(n=220)	(n=307)	(n=307)
Age (y), mean ± SD	70.4 ± 12.1	68.2 ± 12.2	68.3 ± 11.4	68.2 ± 14.1	63.3 ± 14.2	63.5 ± 14.0
Female, sex, n (%)	168 (50.9)	194 (57.9)	119 (53.1)	114 (51.8)	181 (59.0)	180 (58.6)
Race, n (%)^a^						
White	231 (70.0)	202 (60.3)	209 (93.3)	207 (94.1)	191 (62.2)	193 (62.9)
Black	79 (23.9)	115 (34.3)	1 (0.4)	3 (1.4)	12 (3.9)	13 (4.2)
Asian	10 (3.0)	6 (1.8)	3 (1.3)	1 (0.5)	49 (16.0)	48 (15.6)
Native American or Alaska Native	0	1 (0.3)	0	0	32 (10.4)	25 (8.1)
Other	10 (3.0)	11 (3.3)	11 (4.9)	9 (4.1)	23 (7.4)	28 (9.1)
eGFR, mL/min/1.73 m^2^, mean ± SD	23.0 ± 10.8	23.4 ± 11.7	23.0 ± 12.4	22.2 ± 11.0	21.9 ± 11.9	22.5 ± 13.1
Disease history, n (%)						
Diabetes mellitus	224 (67.9)	223 (66.6)	112 (50.0)	110 (50.0)	181 (59.0)	185 (60.3)
Cardiovascular disease	162 (49.1)	168 (50.1)	113 (50.4)	115 (52.3)	100 (32.6)	119 (38.8)
Hemoglobin, g/dL, mean ± SD	9.9 ± 0.8	9.8 ± 0.8	10.9 ± 0.7	10.9 ± 0.7	10.7 ± 0.8	10.7 ± 0.8
Baseline Hb <10.0 g/dL, n (%)	171 (51.8)	178 (53.1)	40 (17.9)	39 (17.7)	61 (19.9)	62 (20.2)
Baseline Hb ≥10.0 g/dL, n (%)	159 (48.2)	157 (46.9)	184 (82.1)	181 (82.3)	246 (80.1)	245 (79.8)
Blood pressure, mm Hg						
Systolic	138.2 (19.2)	137.0 (17.7)	136.0 (15.4)	134.4 (16.7)	136.6 (18.4)	137.1 (17.9)
Diastolic	69.9 (11.5)	71.3 (10.6)	75.8 (10.5)	75.9 (10.4)	75.6 (11.1)	76.2 (10.7)
Supplemental iron use, n (%)						
Not receiving any iron	143 (43.3)	161 (48.1)	102 (45.5)	119 (54.1)	173 (56.4)	178 (58.0)
Receiving oral iron only	172 (52.1)	156 (46.6)	86 (38.4)	63 (28.6)	119 (38.8)	113 (36.8)
Receiving IV iron only	6 (1.8)	11 (3.3)	28 (12.5)	26 (11.8)	9 (2.9)	12 (3.9)
Receiving IV and oral iron	9 (2.7)	7 (2.1)	8 (3.6)	12 (5.5)	6 (2.0)	4 (1.3)
Baseline ESA use						
N	317	324	220	218	295	300
Epoetin, n (%)	227 (71.6)	238 (73.5)	55 (25.0)	57 (26.1)	228 (77.3)	228 (76.0)
Darbepoetin α, n (%)	90 (28.4)	86 (26.5)	130 (59.1)	137 (62.8)	41 (13.9)	50 (16.7)
Methoxy polyethylene glycol-epoetin β, n (%)	–	–	35 (15.9)	24 (11.0)	26 (8.8)	22 (7.3)
Baseline ESA dose						
≤90 U/kg/wk	174 (55.2)	164 (51.4)	182 (83.5)	185 (85.6)	194 (66.9)	209 (69.7)
>90 and <300 U/kg/wk	102 (32.4)	127 (39.8)	35 (16.1)	28 (13.0)	84 (29.0)	83 (27.7)
≥300 U/kg/wk	39 (12.4)	28 (8.8)	1 (0.5)	3 (1.4)	12 (4.1)	8 (2.7)

Abbreviations: ESA, erythropoiesis-stimulating agent; eGFR, estimated glomerular filtration rate; Hb, hemoglobin; IV, intravenous.

a Race and ethnic group were reported by the patient.

Most of the patients in Europe were receiving darbepoetin alfa at baseline (61%), whereas most of the patients were receiving epoetin in the US (72.5%) and non-US/non-Europe groups (76.6%). In Europe, 84.6% of patients were on a low-dose ESA treatment (≤90 U/kg/wk) compared with 53.3% in the US and 68.3% in the non-US/non-Europe groups (Table 1). The mean darbepoetin alfa dose remained relatively stable and low over the course of the study in the European population but was generally higher and more varied in the US and non-US/non-Europe groups throughout the trial (Fig S1, Table S2).

**Figure 1:**
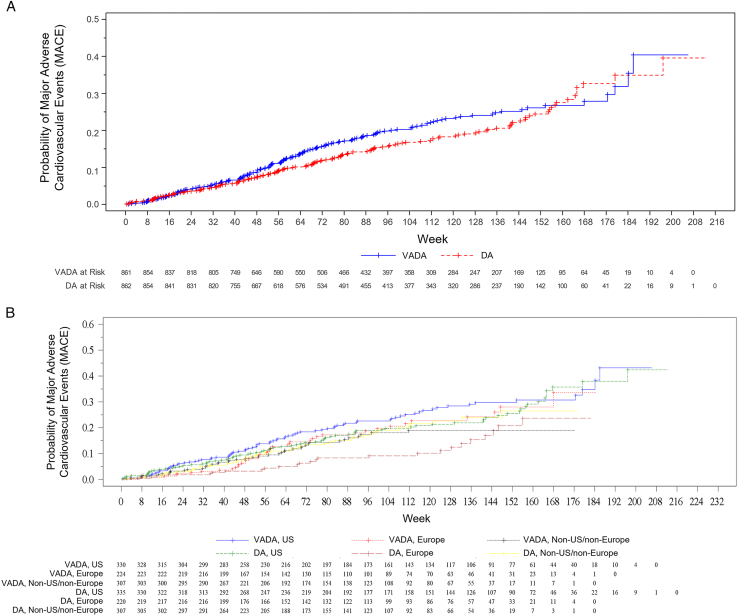
MACE in ESA-treated patients. (A) Total ESA-treated safety population. (B) Region and treatment group. DA, darbepoetin alfa; ESA, erythropoiesis-stimulating agent; MACE, major cardiovascular adverse event; VADA, vadadustat.

In the overall population, the HR for time to first MACE for vadadustat versus darbepoetin alfa was 1.16; 95% CI, 0.93, 1.45 (Fig 1A; Table 2). When stratifying these analyses by region, the HR for MACE was closer to 1 in the US group (1.07; 95% CI, 0.78-1.46) and non-US/non-Europe group (0.91; 95% CI, 0.60-1.37 (Table 2). By contrast, the corresponding HR for MACE was 2.05 (95% CI, 1.24-3.39) in Europe. The *P* value for the interaction between the assigned study treatment and geographical region was marginally significant (*P* = 0.07). Similar trends were seen for expanded MACE (Table S3).

**Table 2:** Cumulative Incidence First MACE in ESA-Treated Patients With NDD-CKD by Regions (Safety Population)

Statistics	Overall		US		Europe		Non-US/non-Europe	
	Vadadustat	Darbepoetin alfa	Vadadustat	Darbepoetin alfa	Vadadustat	Darbepoetin alfa	Vadadustat	Darbepoetin alfa
	n=861	n=862	n=330	n=335	n=224	n=220	n=307	n=307
Cumulative incidence (95% CI)								
52 wk	0.10 (0.08-0.12)	0.08 (0.06-0.10)	0.12 (0.09-0.16)	0.10 (0.07-0.14)	0.08 (0.05-0.13)	0.03 (0.02-0.07)	0.09 (0.06-0.12)	0.09 (0.06-0.13)
104 wk	0.20 (0.17-0.24)	0.17 (0.14-0.20)	0.23 (0.18-0.28)	0.20 (0.16-0.25)	0.20 (0.14-0.27)	0.09 (0.06-0.15)	0.18 (0.14-0.24)	0.19 (0.14-0.25)
156 wk	0.27 (0.23-0.31)	0.26 (0.22-0.31)	0.31 (0.25-0.37)	0.27 (0.22-0.34)	0.28 (0.20-0.38)	0.24 (0.15-0.36)	0.19 (0.14-0.25)	0.27 (0.19-0.36)
208 wk	N/A	0.40 (0.29-0.52)	N/A	0.42 (0.32-0.55)	N/A	N/A	N/A	N/A
Hazard ratio (vadadustat/darbepoetin alfa) (95% CI)	1.16 (0.93, 1.45)		1.07 (0.78, 1.46)		2.05 (1.24, 3.40)		0.91 (0.61, 1.38)	

Abbreviations: CI, confidence interval; ESA, erythropoiesis-stimulating agent; MACE, major adverse cardiovascular event; N/A, not available; NDD-CKD, non–dialysis-dependent chronic kidney disease.

The cumulative incidence of MACE in Europe in the first and second years of the trial was 3% and 9%, respectively, and in the darbepoetin alfa group versus the vadadustat group was 8% and 20%, respectively. The cumulative incidence was similar in the third year of the trial. The cumulative incidence of MACE between the darbepoetin alfa and vadadustat groups within the other regions was similar throughout the trial (Table 2). MACE rates per 100 person-years in the 3 vadadustat groups across regions were 14.5 in the US, 11.6 in Europe, and 10.0 in the non-US/non-Europe groups, whereas event rates in the darbepoetin alfa group were considerably lower in Europe than in the US and non-US/non-Europe groups (6.7 vs 13.3 and 10.5, respectively) (Table 3).

**Table 3:** Summary of Adjudicated MACE in ESA-Treated Patients by Regions (Safety Population)

	Vadadustat		Darbepoetin Alfa	
Overall	n=861 n (%)	PY = 1,580.2 E (E/100 PY)	n=862 n (%)	PY = 1,619.9 E (E/100 PY)
Any MACE	168 (19.5)	195 (12.3)	152 (17.6)	174 (10.7)
All-cause mortality	139 (16.1)	139 (8.8)	139 (16.1)	139 (8.6)
Nonfatal MI	32 (3.7)	37 (2.3)	22 (2.6)	24 (1.5)
Nonfatal stroke	18 (2.1)	19 (1.2)	10 (1.2)	11 (0.7)
US	n=330 n (%)	PY = 674.2 E (E/100 PY)	n=335 n (%)	PY = 700.3 E (E/100 PY)
Any MACE	83 (25.2)	98 (14.5)	77 (23.0)	93 (13.3)
All-cause mortality	63 (19.1)	63 (9.3)	69 (20.6)	69 (9.9)
Nonfatal MI	22 (6.7)	24 (3.6)	15 (4.5)	17 (2.4)
Nonfatal stroke	10 (3.0)	11 (1.6)	7 (2.1)	7 (1.0)
Europe	n=224 n (%)	PY = 395.7 E (E/100 PY)	n=220 n (%)	PY = 415.6 E (E/100 PY)
Any MACE	41 (18.3)	46 (11.6)	25 (11.4)	28 (6.7)
All-cause mortality	38 (17.0)	38 (9.6)	24 (10.9)	24 (5.8)
Nonfatal MI	4 (1.8)	5 (1.3)	3 (1.4)	3 (0.7)
Nonfatal stroke	3 (1.3)	3 (0.8)	1 (0.5)	1 (0.2)
Other	n=307 n (%)	PY = 510.3 E (E/100 PY)	n=307 n (%)	PY = 504.1 E (E/100 PY)
Any MACE	44 (14.3)	51 (10.0)	50 (16.3)	53 (10.5)
All-cause mortality	38 (12.4)	38 (7.4)	46 (15.0)	46 (9.1)
Nonfatal MI	6 (2.0)	8 (1.6)	4 (1.3)	4 (0.8)
Nonfatal stroke	5 (1.6)	5 (1.0)	2 (0.7)	3 (0.6)

Abbreviations: E, events; ESA, erythropoiesis-stimulating agent; MACE, major adverse cardiovascular event; MI, myocardial infarction; PY, person-year.

Examination of the individual events within the composite MACE revealed fewer total deaths (noncardiovascular and cardiovascular causes) reported in Europe in the darbepoetin alfa arm (10.9%; n=24/220) compared with those in the vadadustat arm (17.0%; n=38/224) (Table 3). The causes of death in the European vadadustat group were primarily noncardiovascular or unknown (n=24) when compared with those in the darbepoetin alfa group (n=14). There was no observed excessive imbalance outside Europe and no major imbalance in the cause of death between the groups (Table S4).

To understand if the ESA used before trial participation may have influenced the outcome of the trial, we conducted an exploratory subgroup analysis of MACE in patients receiving darbepoetin alfa at baseline compared with those receiving another ESA at baseline (ie, maintaining darbepoetin alfa vs switching from a different ESA). In the darbepoetin alfa group, the percentage of patients with a MACE in those receiving darbepoetin alfa at baseline was not appreciably different from those on another ESA at baseline (overall, 16.8% vs 17.9%; US, 26.7% vs 21.8%; Europe, 11.7% vs 9.8%; non-US/non-Europe, 14.0% vs 16.8%). In patients randomized to vadadustat from either darbepoetin alfa or another ESA (overall, 17.2% vs 21.0%; US, 23.3% vs 26.0%; Europe, 15.3% vs 23.3%; non-US/non-Europe, 9.8% vs 15.7%), there were fewer MACE in patients who switched from darbepoetin alfa than from another ESA, although this phenomenon was seen only outside the United States.

We adjusted for several baseline characteristics in a post hoc analysis of MACE, including ESA dose and uACR, which did not appreciably change the results of these analyses (Table S5). Post hoc analyses exploring differences in time to first MACE as a function of the interaction between region, treatment, and a number of baseline characteristics found a significant correlation of MACE with baseline disease characteristics that would be expected to correlate with cardiovascular events (age, history of cardiovascular disease, diabetes, and log uACR) in addition to hemoglobin concentration at baseline and Europe (Table S6).

To assess whether the hemoglobin concentration instability caused by switching could explain the differences in MACE, the need for ESA rescue and MACE was explored. In the vadadustat group, ESA rescue was associated with a higher rate of MACE. Overall, 19.1% without ESA rescue and 23.5% with ESA rescue experienced MACE in the vadadustat group. The difference was greater in the Europe region, where 17.9% without ESA rescue and 25.0% with ESA rescue experienced MACE.

With darbepoetin alfa, there was less of an overall relationship with MACE, possibly because of rescue being driven by ≥100% dose increases rather than adding a different ESA. However, in Europe in the darbepoetin alfa group, there was a striking difference in the MACE rate based on ESA rescue: 8.0% without ESA rescue versus 17.1% with ESA rescue (Table S7).

The relationship of ESA rescue to MACE was further explored using the Cox model, accounting for ESA rescue including darbepoetin alfa dose increases of ≥100% occurring before MACE. ESA rescue was a significant covariate when added to the prespecified time to first MACE Cox model, as shown in Table S8. The HR of MACE for ESA narrow rescue (ie, rescue for anemia) was 1.38; 95% CI, 1.04-1.82. This HR was on par with those for baseline hemoglobin concentration, age, sex, cardiovascular history, diabetes, and region, which also were strongly associated with MACE. Adding ESA rescue as a covariate to the prespecified Cox model yielded an HR for MACE of 1.05 (95% CI, 0.83-1.33).

## Discussion

As previously reported, the orally administered HIF-PHI vadadustat met its prespecified safety end point for noninferiority in MACE in patients receiving dialysis in the INNO_2_VATE trials,^13^ but did not meet the same prespecified safety end point in patients not receiving dialysis in the PRO_2_TECT trials.^10^ In the PRO_2_TECT clinical program, 2 separate trials were conducted, 1 in patients with NDD-CKD who were ESA treated (ClinicalTrials.gov identifier: NCT02680574) and another in patients with NDD-CKD who were ESA untreated (ClinicalTrials.gov identifier: NCT02648347), before entering the respective trials. Prespecified individual trial and regional analyses suggested a higher relative risk of MACE for vadadustat versus darbepoetin alfa in regions outside the United States.^10^ The purpose of these analyses were to further investigate potential reasons for these regional differences in the trial of patients with NDD-CKD and anemia previously treated with ESA. For analyses that investigate regional differences in MACE in the PRO_2_TECT trial of ESA-untreated patients, please see our companion article.^12^

In the analyses presented earlier, we showed that when stratified by region, patients who previously were ESA treated and randomized to darbepoetin alfa in Europe showed approximately half the risk of a MACE compared with patients randomized to vadadustat; this was in sharp contrast to participants from the US and non-US/non-Europe groups, where the estimated HR between trial arms was close to the null value. Although it is possible that differences in clinical practice for managing CKD-related anemia among regions increased the risk of geographic heterogeneity in outcomes, these analyses could not establish such a link, but identified marked regional differences in outcomes in the comparator group.

On examining event rates by randomization of group and region, the MACE rates observed in the US group were consistent with previous reports of real-world MACE rates in patients in the United States treated with ESA therapy.^14^ The event rates in the Europe and non-US/non-Europe groups were very similar to the US group, with the notable exception of patients randomized to the darbepoetin alfa group in Europe, in whom the event rates were strikingly lower. In Europe, darbepoetin alfa showed a cumulative incidence less than half of that of vadadustat for the first 2 years, which changed to an incidence that was similar to the other regions and vadadustat in Europe in the third year. There also was an imbalance in the number of noncardiovascular and unknown deaths within Europe, which was not seen in the other regions.

Several demographic characteristic differences were observed among patients in different regions. The trial population enrolled in Europe had a higher proportion of White patients than the non-US/non-Europe trial population, and IV iron use was more common in comparison to the US and non-US/non-Europe groups.

In addition, patients in Europe had a higher percentage of patients on a low-dose ESA (<90 U/kg/wk), and most of the patients showed a hemoglobin concentration at baseline of ≥10 g/dL. Furthermore, most of the patients in Europe were already receiving darbepoetin alfa therapy (vs a different ESA) at baseline compared with those in the non-US/non-Europe and US groups. This suggests that most European patients likely were well controlled on a low dose of darbepoetin alfa at baseline, and continuation of the same ESA therapy (ie, darbepoetin alfa) during a trial, where the comparison group needs to switch and titrate treatment, could reduce a potential risk associated with changing therapies. Therefore, the higher proportion of patients in the darbepoetin alfa arm in Europe who continued receiving their prestudy therapy could have confounded the results, creating an imbalance in the risk of MACE between the 2 trial groups in Europe. This hypothesis is supported by the mean darbepoetin alfa dosing having remained relatively stable over the course of the trial in the European population, but varying in the other populations, a finding that could not be adequately controlled using randomization.

We performed several post hoc analyses to support this hypothesis and found that there was a difference in the MACE in patients who switched from darbepoetin alfa compared with another ESA, but this phenomenon was seen only in Europe. We also tested for an interaction between region and treatment and several baseline characteristics; aside from disease characteristics that would be expected to correlate with a cardiovascular event, baseline hemoglobin concentration was the only significant factor. The limitations of these analyses are the small event numbers in this ESA-treated trial and that this trial was not powered to evaluate region and treatment interactions.

Additional analyses of the relationship of ESA rescue to the risk of MACE suggest that the low rate of MACE in patients who received darbepoetin alfa in Europe may be explained by the baseline characteristics of patients enrolled in this region. The higher proportion of patients receiving darbepoetin alfa and low-dose ESA and with a hemoglobin concentration of ≥10 g/dL is consistent with these patients being more stable at study entry and at low risk of MACE. Only when requiring ESA rescue or converting to vadadustat did these patients experience an increased MACE.

The Trial to Reduce Cardiovascular Events With Aranesp Therapy (TREAT) trial showed lower cardiovascular risk with darbepoetin alfa in patients with diabetes not receiving dialysis in Western Europe/Australia.^15^ More recently, international data from the Dialysis Outcomes and Practice Patterns Study (DOPPS) suggested that the use of long-acting ESAs in patients receiving dialysis showed a positive effect on all-cause mortality in Europe, neither of which was in concordance with other regions.^16^ However, it is possible that the low MACE rate in the darbepoetin alfa arm in Europe happened by chance. The rate was lower than expected from the sample size assumptions and from those observed in the 3 groups that received darbepoetin alfa in patients with NDD-CKD previously untreated with an ESA.^12^ Recently, Singh et al^17^ published their findings that daprodustat was noninferior to darbepoetin alfa with respect to MACE. In the trial by Singh et al^17^, the event rates for darbepoetin alfa were higher than those observed in the European darbepoetin alfa arm of our trial, supporting the idea that the darbepoetin alfa–associated event rate in our trial was unusually low.^17^

In summary, regional differences in time to first MACE were observed in patients with NDD-CKD who were treated with ESA and randomized to receive vadadustat or darbepoetin alfa as part of the PRO_2_TECT trials. Because the low risk of MACE was observed in patients in the darbepoetin alfa arm in Europe, who were generally on low doses of ESA and showed a hemoglobin concentration that was already within target range, it may have been related to a limited need to switch and titrate darbepoetin alfa compared with the non-US/non-Europe group or occurred by chance.
