# Supplementary material for: Safety Endpoints With Vadadustat Versus Darbepoetin Alfa in Patients With Non–Dialysis-Dependent CKD: A Post Hoc Regional Analysis of the PRO2TECT Randomized Clinical Trial of ESA-Treated Patients
Source: Kidney Med. 2023 May 12;5(7):100667. doi: 10.1016/j.xkme.2023.100667 (PMC10329165; doi:10.1016/j.xkme.2023.100667)
Supplement: Supplementary File (PDF) — Figure S1; Table S1-S8. [file mmc1.pdf]

Fig S1. Average weekly dose of study treatment by region (safety population).

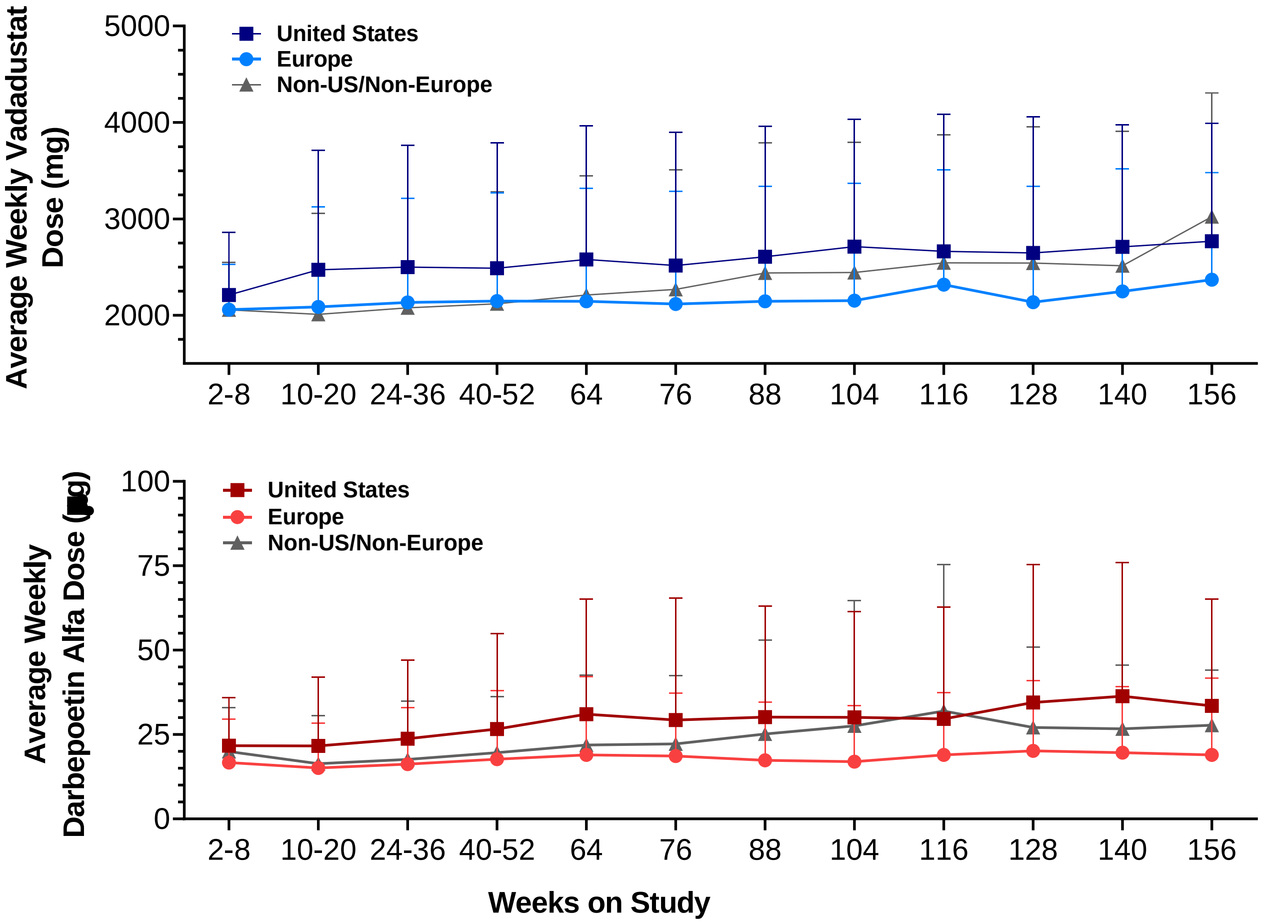

Table S1. Regional Classification of Countries That Enrolled Patients for PRO_2TECT ESA-Treated Trial

**Region**	**US**	**Europe**	**Non-US/non-Europe**
**Countries**	United States of America	Austria	Argentina
		Bulgaria	Australia
		Czech Republic	Brazil
		France	Canada
		Germany	Chile
		Hungary	Colombia
		Italy	Israel
		Poland	Malaysia
		Romania	Mexico
		Serbia	New Zealand
		Slovakia Republic	Russia
		Spain	South Africa
		Turkey	South Korea
		United Kingdom[a]	Ukraine

Abbreviation: ESA, erythropoiesis-stimulating agent.
[a]This trial was conducted while the United Kingdom was part of the European Union.

Table S2. Average Weekly Dose of Study Treatment in ESA-Treated Patients by Region (Safety Population)

	US		Europe		Non-US/non-Europe	
**Study period/visit statistics**	**Vadadustat (n = 330)**	**Darbepoetin Alfa (n = 335)**	**Vadadustat (n = 224)**	**Darbepoetin alfa (n = 220)**	**Vadadustat (n = 307)**	**Darbepoetin alfa (n = 307)**
**Weeks 2–8**						
N	330	335	224	220	307	307
Mean (SD)	2210.1 (650.1)	21.7 (14.3)	2058.4 (466.6)	16.7 (12.9)	2055.7 (490.9)	20.0 (13.1)
Median	2266.7	16.4	2100.0	13.5	2100.0	18.1
Q1, Q3	1966.7, 2683.3	10.6, 28.5	1826.9, 2216.7	9.1, 20.0	1783.3, 2333.3	11.5, 25.0
**Weeks 10–20**						
N	302	319	214	214	284	296
Mean (SD)	2471.3 (1239.1)	21.6 (20.4)	2087.2 (1037.9)	15.1 (13.3)	2009.6 (1047.7)	16.4 (14.2)
Median	2475.0	15.4	2100.0	10.7	2100.0	12.7
Q1, Q3	1453.9, 3565.4	8.0, 27.3	1130.8, 2907.7	5.3, 20.0	1050.0, 2728.9	6.9, 20.7
**Weeks 24–36**						
N	268	335	200	206	263	282
Mean (SD)	2499.0 (1262.5)	23.8 (23.3)	2133.2 (1079.4)	16.2 (16.7)	2078.6 (1136.2)	17.6 (17.3)
Median	2498.4	16.6	2100.0	10.0	2100.0	13.1
Q1, Q3	1331.3, 3698.4	9.6, 28.7	1050.0, 3150.0	5.0, 20.0	1050.0, 2987.6	7.5, 20.5
**Weeks 40–52**						
N	225	249	162	174	206	217
Mean (SD)	2488.7 (1299. 0)	26.7 (28.3)	2147.4 (1122.8)	17.7 (20.3)	2119.1 (1160.7)	19.7 (16.6)
Median	2355.0	20.0	2100.0	10.9	2100.0	15.0
Q1, Q3	1380.0, 3780.0	10.0, 30.1	1050.0, 3150.0	5.0, 20.0	1050.0, 3150.0	9.0, 25.0

Abbreviation: ESA, erythropoiesis-stimulating agent; SD, standard deviation.
Note: The unit for vadadustat is mg, and the unit for darbepoetin alfa is µg. For each visit, any dosing data within the analysis window are included for the summary.

Table S3. Time to First Expanded MACE and All-Cause Mortality in ESA-Treated Patients by Region (Safety Population)

	**Hazard ratio (vadadustat/darbepoetin alfa) (95% confidence interval)**			
**Endpoint**	**Overall**	**US**	**Europe**	**Non-US/non-Europe**
MACE+ (plus hospitalization for heart failure, or thromboembolic event excluding vascular access thrombosis)	1.04 (0.85, 1.27)	0.95 (0.72, 1.25)	1.58 (1.02, 2.44)	0.92 (0.62, 1.36)
All-cause mortality	1.04 (0.82, 1.32)	0.90 (0.64, 1.27)	1.92 (1.15, 3.23)	0.82 (0.54, 1.270)

Abbreviations: ESA, erythropoiesis-stimulating agent; MACE, major adverse cardiovascular event.

Table S4. Summary of Primary Cause of Death in NDD-CKD ESA-Treated Patients by Region (Safety Population)

	US N (%)/(per 100 PY)		Europe N (%)/(per 100 PY)		Non-US/non-Europe N (%)/(per 100 PY)	
**Primary cause of death**	**VADA N=330/ PY=674.2**	**DA N=335/ PY=700.3**	**VADA N=224/ PY=395.7**	**DA N=220/ PY=415.6**	**VADA N=307/ PY=510.3**	**DA N=307/ PY=504.1**
CV death	26 (7.9%)/(3.9)	32 (9.6%)/(4.6)	14 (6.3%)/(3.5)	10 (4.5%)/(2.4)	16 (5.2%)/(3.1)	24 (7.8%)/(4.8)
Sudden death	10 (3.0%)/(1.5)	6 (1.8%)/(0.9)	3 (1.3%)/(0.8)	3 (1.4%)/(0.7)	7 (2.3%)/(1.4)	5 (1.6%)/(1.0)
Presumed CV death	3 (0.9%)/(0.4)	9 (2.7%)/(1.3)	3 (1.3%)/(0.8)	2 (0.9%)/(0.5)	3 (1.0%)/(0.6)	6 (2.0%)/(1.2)
Pump failure	7 (2.1%)/(1.0)	8 (2.4%)/(1.1)	2 (0.9%)/(0.5)	1 (0.5%)/(0.2)	2 (0.7%)/(0.4)	5 (1.6%)/(1.0)
Fatal stroke	2 (0.6%)/(0.3)	3 (0.9%)/(0.4)	3 (1.3%)/(0.8)	0 (0%)/(0)	4 (1.3%)/(0.8)	2 (0.7%)/(0.4)
Fatal MI	3 (0.9%)/(0.4)	4 (1.2%)/(0.6)	2 (0.9%)/(0.5)	1 (0.5%)/(0.2)	0 (0%)/(0)	4 (1.3%)/(0.8)
Other CV	0 (0%)/(0)	1 (0.3%)/(0.1)	1 (0.4%)/(0.3)	0 (0%)/(0)	0 (0%)/(0)	0 (0%)/(0)
Presumed sudden death	0 (0%)/(0)	0 (0%)/(0)	0 (0%)/(0)	3 (1.4%)/(0.7)	0 (0%)/(0)	2 (0.7%)/(0.4)
CV procedural	1 (0.3%)/(0.1)	1 (0.3%)/(0.1)	0 (0%)/(0)	0 (0%)/(0)	0 (0%)/(0)	0 (0%)/(0)
Non-CV death	26 (7.9%)/(3.9)	29 (8.7%)/(4.1)	17 (7.6%)/(4.3)	14 (6.4%)/(3.4)	18 (5.9%)/(3.5)	19 (6.2%)/(3.8)
Renal	11 (3.3%)/(1.6)	8 (2.4%)/(1.1)	3 (1.3%)/(0.8)	3 (1.4%)/(0.7)	3 (1.0%)/(0.6)	5 (1.6%)/(1.0)
Infection	3 (0.9%)/(0.4)	4 (1.2%)/(0.6)	8 (3.6%)/(2.0)	6 (2.7%)/(1.4)	8 (2.6%)/(1.6)	6 (2.0%)/(1.2)
GI	1 (0.3%)/(0.1)	1 (0.3%)/(0.1)	1 (0.4%)/(0.3)	1 (0.5%)/(0.2)	3 (1.0%)/(0.6)	2 (0.7%)/(0.4)
Other non-CV	4 (1.2%)/(0.6)	3 (0.9%)/(0.4)	0 (0%)/(0)	1 (0.5%)/(0.2)	2 (0.7%)/(0.4)	1 (0.3%)/(0.2)
Malignancy	1 (0.3%)/(0.1)	9 (2.7%)/(1.3)	4 (1.8%)/(1.0)	3 (1.4%)/(0.7)	2 (0.7%)/(0.4)	4 (1.3%)/(0.8)
Pulmonary	5 (1.5%)/(0.7)	3 (0.9%)/(0.4)	0 (0%)/(0)	0 (0%)/(0)	0 (0%)/(0)	1 (0.3%)/(0.2)
Accidental	1 (0.3%)/(0.1)	1 (0.3%)/(0.1)	1 (0.4%)/(0.3)	0 (0%)/(0)	0 (0%)/(0)	0 (0%)/(0)
Unknown	11 (3.3%)/(1.6)	8 (2.4%)/(1.1)	7 (3.1%)/(1.8)	0 (0%)/(0)	4 (1.3%)/(0.8)	3 (1.0%)/(0.6)

Abbreviations: CV, cardiovascular; DA, darbepoetin alfa; ESA, erythropoiesis-stimulating agent; GI, gastrointestinal; MI, myocardial infarction; NDD-CKD, non–dialysis-dependent chronic kidney disease; PY, patient-year; VADA, vadadustat.

Table S5. Cox Model Analyses of Baseline Variables and Time to MACE in ESA-Treated Patients With NDD-CKD (Safety Population)

Factor	Overall HR (95% CI)	US HR (95% CI)	Europe HR (95% CI)	Non-US/non-Europe HR (95% CI)
Primary MACE	1.16 (0.930, 1.446)	1.07 (0.782, 1.461)	2.05 (1.237, 3.392)	0.91 (0.607, 1.374)
Primary MACE by adding baseline LDL cholesterol (Calculated)	1.17 (0.927, 1.465)	1.05 (0.758, 1.451)	2.05 (1.203, 3.483)	0.99 (0.653, 1.510)
Primary MACE by adding aspirin dose group at baseline	1.17 (0.936, 1.459)	1.07 (0.781, 1.464)	2.09 (1.264, 3.464)	0.92 (0.607, 1.385)
Primary MACE by adding weight	1.12 (0.893, 1.397)	1.01 (0.734, 1.387)	2.07 (1.238, 3.446)	0.88 (0.585, 1.337)
Primary MACE by adding ESA type at baseline	1.17 (0.941, 1.464)	1.08 (0.787, 1.478)	2.05 (1.232, 3.424)	0.93 (0.619, 1.407)
Primary MACE by adding ESA dose group at baseline	1.15 (0.924, 1.438)	1.08 (0.790, 1.478)	2.02 (1.214, 3.369)	0.92 (0.608, 1.384)
Primary MACE by ESA type and ESA dose at baseline	1.17 (0.938, 1.460)	1.06 (0.772, 1.454)	2.08 (1.244, 3.471)	0.94 (0.622, 1.416)
Primary MACE by adding baseline uACR group	1.10 (0.871, 1.386)	1.00 (0.720, 1.400)	2.12 (1.232, 3.645)	0.82 (0.534, 1.251)
Primary MACE by adding log (uACR) at baseline	1.08 (0.853, 1.359)	0.99 (0.709, 1.380)	2.04 (1.182, 3.527)	0.80 (0.522, 1.222)
Primary MACE by adding log (uACR) and eGFR at baseline	1.08 (0.857, 1.365)	0.99 (0.706, 1.375)	2.21 (1.266, 3.855)	0.80 (0.522, 1.223)

Abbreviations: CI, confidence interval; eGFR, estimated glomerular filtration rate; ESA, erythropoiesis-stimulating agent; HR, hazard ratio; LDL, low-density lipoprotein; MACE, major adverse cardiovascular event; NDD-CKD, non–dialysis-dependent chronic kidney disease; uACR, urine albumin-to-creatinine ratio.
Note: MACE includes all-cause mortality, nonfatal myocardial infarction, or nonfatal stroke. MACE occurring at any time following the first dose and prior to each subject's end of study date are included.

Table S6. Time to First MACE—Interaction of Region and Treatment by Baseline Parameters in ESA-Treated Patients With NDD-CKD (Safety Population)

**Parameter**	**Comparator Groups**	**Overall N = 1723 *P* value**
Actual treatment in vadadustat 300 mg	Darbepoetin alfa	0.8806
Baseline hemoglobin (g/dL)	Continuous variable[a]	0.0275
Age	Continuous variable[a]	<0.0001
Female sex	Male	0.0171
Race non-White	White	0.1591
No CV disease history (CAD, MI, stroke, HF)	Yes	<0.0001
No history of diabetes	Yes	0.0136
NYHA class 0 or I	NYHA class II or III	0.9264
Region		
Europe	United States	0.1309
Non-US/non-Europe		0.2652
Baseline LDL cholesterol (mg/dL)	Continuous variable[a]	0.8826
Baseline aspirin ≤300 mg	Baseline aspirin >300 mg	0.6713
Baseline weight (kg)	Continuous variable[a]	0.3440
Baseline ESA medication type		
Darbepoetin alfa	Methoxy polyethylene glycol-epoetin beta	0.4091
Epoetin		0.3068
Baseline ESA dose		
≤90 U/kg/week	≥300 U/kg/week	0.6071
>90 and <300 U/kg/week		0.5029
Baseline log uACR	Continuous variable[a]	0.0011
Baseline eGFR (mL/min/1.73 m^2)	Continuous variable[a]	0.1834

Abbreviations: CAD, coronary artery disease; CV, cardiovascular; eGFR, estimated glomerular filtration rate; ESA, erythropoiesis-stimulating agent; HF, heart failure; LDL, low-density lipoprotein; MACE, major adverse cardiovascular event; MI, myocardial infarction; NDD-CKD, non–dialysis-dependent chronic kidney disease; NYHA, New York Heart Association; uACR, urine albumin-to-creatinine ratio.
Note: MACE includes all-cause mortality, nonfatal MI, or nonfatal stroke. MACE occurring at any time following the first dose and prior to each subject's end of study date are included.
[a]Represents a continuous variable with no discrete categories to compare against.

Table S7. MACE in NDD-CKD ESA-Treated Patients by Narrow Rescue[a] and ≥100% Darbepoetin Alfa Dose Increase

		Vadadustat		Darbepoetin alfa	
**Region**	**Narrow Rescue[a]**	**Subject N**	**MACE No. of Events (%)**	**Subjects N**	**MACE No. of Events (%)**
Europe	No	212	38 (17.9)	138	11 (8.0)
	Yes	12	3 (25.0)	82	14 (17.1)
ROW	No	288	41 (14.2)	203	36 (17.7)
	Yes	19	3 (15.8)	104	14 (13.5)
US	No	276	69 (25.0)	180	49 (27.2)
	Yes	54	14 (25.9)	155	28 (18.1)
Overall	No	776	148 (19.1)	521	96 (18.4)
	Yes	85	20 (23.5)	341	56 (16.4)

Abbreviations: ESA, erythropoiesis-stimulating agent; MACE, major adverse cardiovascular event; NDD-CKD, non–dialysis-dependent chronic kidney disease; ROW, rest of world.
[a]Narrow rescue: rescue for worsening anemia with ESA medication or darbepoetin alfa dose increase ≥100% from the prior dose before MACE.

Table S8. Time to First MACE in NDD-CKD ESA-Treated Patients With *P* Values and Hazard Ratios, Adding Narrow Rescue[a]

Parameter	*P* value	HR	95% CI	
Treatment: vadadustat vs darbepoetin alpha	0.69	1.05	0.83	1.33
Baseline Hb (g/dL)	<0.001	0.77	0.67	0.88
Age (<65 vs ≥65)	<0.001	0.46	0.35	0.60
Female vs male (reference)	<0.01	0.72	0.58	0.90
Pooled race group 1 (all others vs White)	0.06	1.29	0.99	1.69
CV disease history (CAD, MI, stroke, HF) (no vs yes)	<0.001	0.50	0.39	0.63
History of DM (no vs yes)	0.02	0.75	0.59	0.96
Strat 02 NYHA class (0 or I) vs (II or III)	0.92	0.98	0.73	1.33
Strat 03 region Europe vs US	0.27	0.85	0.64	1.14
Strat 03 region ROW vs US	0.04	0.72	0.52	0.99
ESA rescue (narrow) and 100% darbepoetin alpha increase (yes vs no)	0.03	1.38	1.04	1.82

Abbreviations: CAD, coronary artery disease; CI, confidence interval; CV, cardiovascular, DM, diabetes mellitus, ESA, erythropoiesis-stimulating agent; Hb, hemoglobin, HF, heart failure; HR, hazard ratio; MACE, major adverse cardiovascular event, MI, myocardial infarction; NDD-CKD, non–dialysis-dependent chronic kidney disease; NYHA, New York Heart Association; ROW, rest of world.
[a]Narrow rescue: rescue for worsening anemia with ESA medication or darbepoetin alfa dose increase ≥100% from the prior dose before MACE.
